# Microcirculation Approach in HELLP Syndrome Complicated by Posterior Reversible Encephalopathy Syndrome and Massive Hepatic Infarction

**DOI:** 10.1155/2014/389680

**Published:** 2014-11-18

**Authors:** Stephanno Gomes Pereira Sarmento, Eduardo Feliz Martins Santana, Felipe Favorette Campanharo, Edward Araujo Júnior, Flavia Ribeiro Machado, Nelson Sass, Antonio Fernandes Moron

**Affiliations:** ^1^Department of Obstetrics, Paulista School of Medicine, Federal University of São Paulo (EPM-UNIFESP), Rua Carlos Weber 956, Apartamento 113 Visage, 05303-000 São Paulo, SP, Brazil; ^2^Department of Medicine, Paulista School of Medicine, Federal University of São Paulo (EPM-UNIFESP), 05303-000 São Paulo, SP, Brazil

## Abstract

HELLP syndrome is a complication of severe forms of preeclampsia and occurs mainly in the third trimester of pregnancy. In extreme cases, it may evolve unfavorably and substantially increase maternal mortality. We present the case of an 18-year-old pregnant woman who was admitted to our emergency service in her 31st week, presenting with headache, visual disturbances, and epigastralgia, with progression to a severe condition of HELLP syndrome followed by posterior reversible encephalopathy syndrome (PRES) and hepatic infarction. We highlight the approach taken towards this patient and the case management, in which, in addition to the imaging examinations routinely available, we also used the sidestream dark field (SDF) technique to evaluate the systemic microcirculation.

## 1. Introduction

Preeclampsia generally affects pregnant women in their third trimester and is classically characterized by elevation of pressure and proteinuria levels. HELLP syndrome (hemolysis, elevated liver enzymes, and low platelets) is a complication of severe forms of preeclampsia that compromises the blood system, with hemolysis, hepatic lesions, and low platelet counts [1, 2]. Its incidence is approximately one to two cases per 1000 pregnancies and reaches 5% among women with preeclampsia [3]. In extreme cases, it may result in hepatic infarction and posterior reversible encephalopathy syndrome (PRES), which is defined as a predominantly vasogenic form of cerebral edema of parietooccipital location that is typically reversible, with variable clinical presentation. It is not exclusively found in preeclampsia cases [4].

Techniques that directly evaluate the perfusion of the microcirculation at the bedside have been developed to complement the traditional macrohemodynamic parameters. These techniques have been tested in different clinical situations such as shock and sepsis.

Here, we describe a case of HELLP syndrome that severely affected multiple systems, in which we emphasize the use of imaging diagnostic techniques in association with the sidestream dark field (SDF) technique on the microcirculation [5].

## 2. Case Presentation

The patient was a single black 18-year-old woman who was a student born and living in São Paulo, Brazil. She was primiparous and in her second pregnancy, without complications in her previous pregnancy. She had not attended prenatal care consultations. Her personal history included traces of sickle cell disease. She was admitted to the Emergency Service of Hospital São Paulo, Paulista School of Medicine, Federal University of São Paulo (EPM-UNIFESP) with a complaint of high-intensity holocranial headache in association with blurring of vision and pain in the epigastric region, focused on a narrow band, which had started on the preceding day. There was a report that the patient had had an episode of convulsion during the previous night and another episode while being brought to the hospital.

During the initial attendance, the patient was conscious and presented with arterial pressure of 140/90, heart rate of 80 bpm, equally photoreactive pupils, agitation, spatial orientation, temporal disorientation, muscle disorders, markedly diminished visual acuity, edema of ++/4+, uterine height of 25 cm, normal uterine tonus, normal heartbeats, and absence of uterine dynamics.

**Figure 1:**
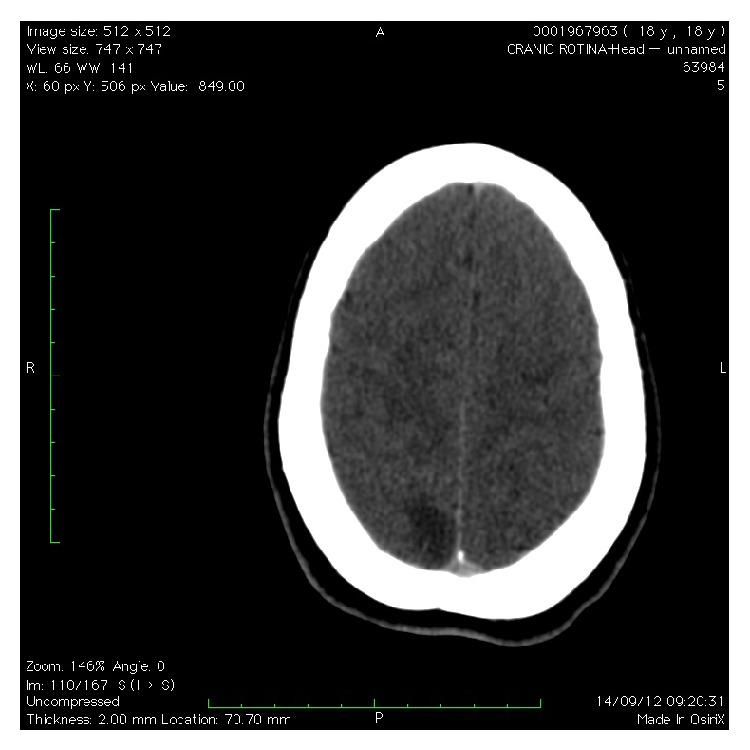
Cranial tomography showing cerebral parenchyma with bilateral occipital subcortical hypoattenuating areas associated with a slight expansive effect that extends anteriorly towards the parietal regions, which do not change after injection of contrast medium, with diffusely diminished cerebral sulci. Alterations compatible with bilateral occipital areas of subcortical vascular disorder, observed in patients with hypertensive encephalopathy, as observed in cases of preeclampsia known as posterior reversible leukoencephalopathy.

**Figure 2:**
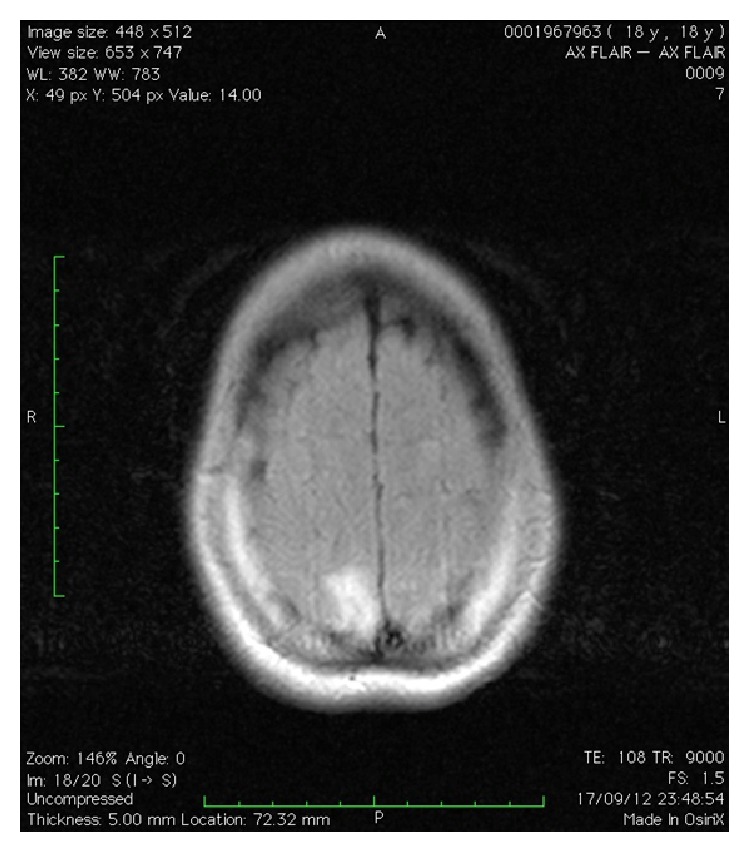
Cranial angioresonance showing cerebral parenchyma with bilateral occipital subcortical areas that present hypersignal in FLAIR and T2, which are associated with a slight expansive effect and do not change after injection of contrast medium, with diffusely compressed cerebral sulci. Alterations compatible with bilateral occipital areas of subcortical vascular disease, which may be present in patients with hypertensive encephalopathy, as observed in cases of preeclampsia known as posterior reversible leukoencephalopathy.

In the admission room, the ocular fundus was examined, showing papilledema. An ultrasound examination showed a pregnancy of 31 weeks, fetal growth restriction with normal Doppler velocimetry, and oligohydramnios. Ophthalmic artery Doppler showed a peak ratio of 0.88. Cranial tomography was performed with contract medium (Figure 1) and was subsequently complemented with cranial angioresonance imaging (Figure 2), showing a bilateral occipital hypoattenuating area that also reached the parietal region, without respecting anatomical divisions and without corresponding to cerebral sulci or presenting any mass effect. This finding was suggestive of PRES, with cortical blindness. The laboratory tests produced the following results: Hb 13.4 g/dL; Ht 41.7%; platelets: 174,000/uL; creatinine: 1.23 mg/dL; urea: 31 mg/dL; total bilirubin: 0.89 mg/dL; TGO 188 U/L; TGP 131 U/L; DHL 503 U/L; proteinuria in a single sample < 0.15 g/L, normal coagulogram, normal biochemistry of cerebrospinal fluid, and culturing without abnormalities; and serological tests negative (HIV, VDRL, hepatitis B and C, toxoplasmosis, rubella, and cytomegalovirus).

In the light of the initial hypotheses of eclampsia, partial HELLP syndrome, and PRES, monitored treatment with magnesium sulphate, hydralazine, and corticoids was started in the intensive care unit. The patient evolved with worsening of the symptoms and peak pressure, and it was therefore decided to conclude the pregnancy through cesarean section, with postoperative care in an intensive care unit. The newborn was female, weighing 1670 g, with Apgar scores of 3 in the first minute and 8 in the fifth minute; the placenta weighed 340 g.

**Figure 3:**
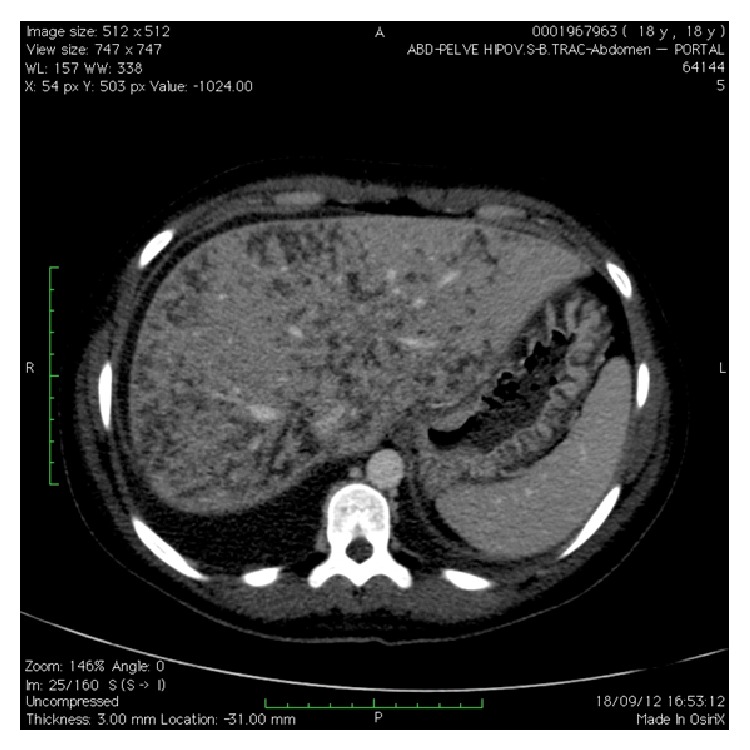
Magnetic resonance imaging on the upper abdomen showing liver with slightly increased dimensions and a regular outline, presenting multiple irregular hypodense areas of serpiginous type that affect the entire liver, without highlighting from the contrast, thus corresponding to areas of hepatic infarction.

On the first and second postoperative days, the cortical blindness improved, but the band of abdominal pain continued, with worsening of the laboratory tests: Hb 6.8 g/dL; Ht 20.4%; platelets: 128,000/uL; creatinine: 1.16; urea: 36 mg/dL; hemolysis and elevation of hepatic transaminases; normal coagulogram; and proteinuria: 1.22 g/24 h. Because of the abdominal symptoms, resonance imaging of the upper abdomen was requested (Figure 3), which was subsequently complemented with cholangiopancreatography.

**Figure 4:**
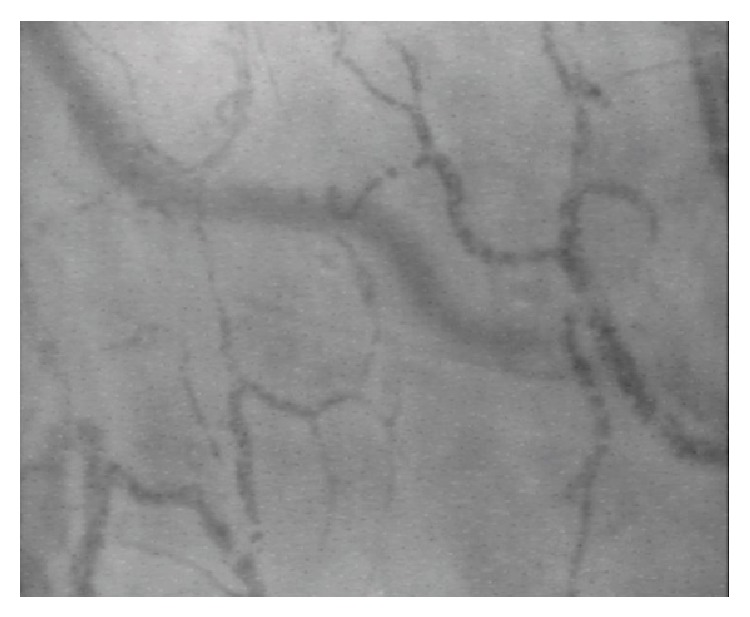
Sidestream dark field images of the sublingual microcirculation. The damage to the endothelial cell breaks the microvascular chain and potentially impedes sufficient tissue perfusion area.

On the second postoperative day, the microcirculation was evaluated by means of the SDF technique (Figure 4) through the oral mucosa. It was observed that the impairment of the vessels was coherent with a condition of systemic endothelial lesion. However, this assessment was made after hospitalization, with the condition of decompensation already advanced.

The patient was kept in hospital for 14 days and, over this period, all the laboratory tests and imaging examinations became normal again. The patient was then released and outpatient follow-up was maintained.

## 3. Discussion

The clinical presentation of PRES may be similar to that of hypertensive encephalopathy, with nonspecific findings and involvement of the white matter, particularly in the occipital region. The etiology remains unknown, although it is probably multifactorial and involves action by circulating cytotoxic factors, thus leading to disorders of cerebral self-regulation, increased vascular permeability, and vasogenic edema [6]. Occurrences of convulsions, headache, and visual disturbances are found in 62.5%, 58%, and 50% of the cases, respectively. PRES relating to preeclampsia follows a course with a larger number of cerebral areas affected, but because it occurs in young women with less comorbidity, it tends to present better evolution and better reversibility than seen in PRES due to other causes [7].

Hepatic infarction is another severe and infrequent complication in cases of HELLP syndrome and has been associated with death in 16% of the patients [8]. It should be considered to be a systemic process and not just a primary arterial disease [9]. It is believed that the ligand CD95, which is a humoral factor derived from the placenta that correlates with the pathogenesis of HELLP syndrome, mediates an increased response to apoptosis of hepatocytes together with cytotoxic activity [10]. Computed tomography is an important tool for making the differential diagnosis of hepatic dysfunctions of pregnancy, but published studies on tomographic findings from hepatic lesions in HELLP syndrome are still scarce [11].

Evaluation of the microcirculation is usually done by means of laboratory parameters such as serum assaying of arterial lactate and central venous oxygen saturation. These parameters only allow an overall estimate of the oxygenation of organ tissues and do not evaluate the exact location where the exchange of nutrients and oxygen with the tissues takes place.

The SDF device captures images that highlight the microcirculation by means of emission of green light into the tissues (reaching a depth of approximately 3 mm) and absorption of this light by hemoglobin. Through reading what is reflected by the tissue, it becomes possible to identify the structures that make up the microcirculation [12]. Through using this method, several studies have shown that there are significant changes to the microcirculation in a variety of clinical situations and that these changes have a direct association with organ dysfunctions and death [13, 14]. Images can be obtained from several tissue surfaces, but the site usually evaluated in clinical studies is the sublingual region. The importance of this region relates to its embryological origin, which is similar to the splanchnic circulation and is closely related to tissue perfusion, given that in situations of hypoperfusion, this is the region with the greatest relationship of dependence on adequate blood flow.

The analysis on the microcirculation can be reliably accomplished by using semiquantitative scoring, and the interpretation should be done through acquisition of three good-quality video sequences of at least 20 seconds each that avoid artifacts. Absence of or diminished percussion in large vessels suggests that a pressure artifact is present. The circulation velocity inside the larger vessels forms the reference point for velocity analysis in the capillary vessels. These are interpreted by the software according to their perfusion, heterogeneity, density, and the quantities in the quadrants of each video. Specific indices are calculated for the analysis.

Much doubt still remains in the literature with regard to the direct relationship, in which the degree of change to the microcirculation would provide precise determination of the unfavorable maternal-fetal morbidity-mortality outcome, and the extent to which this method is superior to the indirect methods that are generally used.

Hypertensive alterations during pregnancy are important causes of maternal death and are becoming increasingly prevalent. It is imperative to pay greater attention to doctors' training, particularly among those working in emergency services, so as to ensure that they can rapidly identify preeclampsia, eclampsia, and HELLP syndrome and to ensure that treatments for pregnant women and complete investigations of possible damage to target organs are optimized. When faced with severe complications, all diagnostic means should be used: it is likely that through adding analysis on microcirculation to the propaedeutics of hypertension, identification of these unfavorable events and structuring of better prenatal follow-up will become more effective.
